# Surgical Intensity and Survival After Surgery for Extradural Spinal Metastases: Mechanical Instability, Tumor Biology, and Systemic Reserve

**DOI:** 10.3390/cancers18152512

**Published:** 2026-08-05

**Authors:** Aydin Talat Baydar, Muhammed Bayindir, Aysenur Coskun Baydar, Baran Taskala

**Affiliations:** 1 Department of Neurosurgery, Basaksehir Cam and Sakura City Hospital, Istanbul 34480, Türkiye; 2 Department of Surgical Nursing, Faculty of Health Sciences, Istanbul Atlas University, Istanbul 34408, Türkiye

**Keywords:** spinal metastases, surgical intensity, spinal metastasis invasiveness index, 90-day mortality, overall survival, mechanical instability

## Abstract

**Background/Objectives:** Surgery for extradural spinal metastases must balance neurologic and mechanical goals against limited survival. We evaluated whether a predefined three-tier surgical-intensity classification aligned with established measures of operative burden and whether intensity was associated with 90-day mortality or overall survival. **Methods:** This retrospective cohort included 141 adults with extradural spinal metastases operated on between June 2020 and December 2025. Surgical intensity was classified as low, moderate, or high. SMII was calculated for all patients as a secondary convergent construct measure. Firth penalized logistic regression and Cox regression adjusted for performance status, albumin, and tumor biology. **Results:** There were 37 low-, 39 moderate-, and 65 high-intensity procedures. Median SMII increased from 6 [5–11] to 14 [11.5–18.5] and 23 [18–28] across the three groups (*p* < 0.001; Spearman ρ = 0.711). High-intensity surgery was concentrated among unstable SINS lesions and was associated with longer operative time, transfusion, and hospitalization. Ninety-day mortality was 24.3%, 12.8%, and 23.1% for low-, moderate-, and high-intensity procedures, respectively. High intensity was not statistically associated with 90-day mortality (adjusted OR 0.81, 95% CI 0.28–2.36) or overall survival (adjusted HR 1.08, 95% CI 0.65–1.80). ECOG ≥ 3 and aggressive solid/unknown tumor biology were associated with worse outcomes. **Conclusions:** The intensity classification showed strong convergent validity with SMII and perioperative burden. Within this surgically selected cohort, intensity was not independently associated with early mortality or overall survival, although clinically meaningful differences cannot be excluded. Operative magnitude should be interpreted alongside mechanical need, systemic reserve, and tumor biology.

## Simple Summary

Operations for spinal metastases range from decompression alone to long-segment fixation, corpectomy, and circumferential reconstruction. The magnitude of surgery must be considered together with mechanical instability, tumor biology, and the patient’s systemic condition. We studied 141 adults who underwent surgery for extradural spinal metastases and compared a clinically defined three-tier surgical-intensity classification with the established Spinal Metastasis Invasiveness Index (SMII). SMII increased markedly from low- to moderate- to high-intensity surgery, supporting the classification as a measure of operative magnitude. Higher intensity was also associated with greater instability, longer operative duration, more transfusion, and longer hospitalization. After adjustment, however, surgical intensity was not statistically associated with 90-day mortality or overall survival; poor performance status and aggressive tumor biology showed stronger associations. The postoperative survival thresholds reported here are descriptive and do not prove quality-of-life benefit or appropriateness. The findings support individualized multidisciplinary selection rather than judging an operation by size alone.

## 1. Introduction

The management of extradural spinal metastases has evolved from a binary choice between radiotherapy and decompression into a multidisciplinary process integrating neurologic compression, tumor radiosensitivity, mechanical instability, systemic disease burden, expected survival, and patient goals. Surgery is usually palliative, but it may preserve ambulation, restore or maintain neurologic function, relieve mechanical pain, stabilize the spine, and enable subsequent oncologic treatment. The randomized trial conducted by Patchell et al. established the role of decompressive surgery followed by radiotherapy in selected patients with metastatic epidural spinal cord compression [1]. Contemporary decision-making, however, is not defined by epidural compression alone.

The NOMS framework formalized four interdependent domains: neurologic, oncologic, mechanical, and systemic [2]. Within this framework, the Spinal Instability Neoplastic Score (SINS) provides a standardized language for tumor-related mechanical instability [3,4], whereas the Bilsky epidural spinal cord compression scale describes epidural disease severity [5]. These instruments improve communication and selection but do not prescribe the magnitude of an operation. Depending on anatomy and treatment goals, surgery may consist of decompression alone, short-segment fixation, long-segment reconstruction, corpectomy, anterior column reconstruction, cement augmentation, or circumferential surgery.

This variation creates a recurring dilemma: does the magnitude of surgery itself contribute materially to early mortality, or does it primarily reflect the mechanical problem for which surgery was selected? Short postoperative survival landmarks, particularly 30 and 90 days, remain relevant to perioperative counseling and treatment planning. They are nevertheless descriptive time horizons rather than validated measures of appropriateness or patient-centered benefit.

Multiple prognostic systems estimate survival after surgery for spinal metastases. Tomita and Tokuhashi linked tumor biology and systemic disease to surgical strategy [6,7]. The New England Spinal Metastasis Score and SORG models incorporated broader clinical, laboratory, and oncologic inputs [8,9,10,11,12,13], and recent temporal and external validations confirm that survival estimation remains necessary despite advances in systemic therapy [14,15]. Contemporary population-based and institutional studies continue to report substantial early and 1-year mortality, with outcomes influenced by tumor type, performance status, systemic disease, nutritional reserve, and ambulatory function [16,17,18,19,20,21]. A recent national surgical-quality cohort likewise identified dependent status, urgency, albumin, and surgical complexity as correlates of short-term adverse outcomes [22].

Procedure-related burden can be quantified using the Spinal Metastasis Invasiveness Index (SMII), which was developed to estimate operative duration and blood loss from weighted operative components, tumor vascularity, and embolization status [23]. By contrast, clinically recognizable intensity tiers may better communicate the overall reconstructive strategy. Minimally invasive and hybrid approaches can reduce selected perioperative burdens, although comparative evidence remains heterogeneous [24,25], and separation surgery illustrates how operative extent is increasingly integrated with postoperative radiation rather than pursued as an isolated definitive intervention [26,27,28]. Prospective multicenter studies also demonstrate that patient-reported outcomes and quality of life can improve after appropriately selected surgery, emphasizing that survival alone is an incomplete measure of benefit [29,30].

We therefore evaluated a predefined three-tier surgical-intensity classification in a contemporary cohort of adults undergoing surgery for extradural spinal metastases. The objectives were to determine whether the classification aligned with SINS-defined instability, perioperative burden, and SMII, and whether surgical intensity was associated with 90-day mortality or overall survival after accounting for systemic reserve and tumor biology. We hypothesized that intensity would show strong convergent validity with mechanical and procedure-related burden, whereas survival would be associated more consistently with performance status and tumor biology.

## 2. Materials and Methods

### 2.1. Study Design and Reporting

This retrospective observational cohort study was conducted at a high-volume tertiary referral center. We reviewed all adults who underwent surgery for spinal metastatic disease between June 2020 and December 2025. Eligible cases were identified from institutional surgical records, and the clinical record for each case was reviewed using the electronic medical record, operative notes, pathology reports, imaging studies, laboratory results, and follow-up documentation. The patient was the unit of analysis, and each patient contributed one index surgical treatment episode. Baseline variables were defined from information available at or before the index operation. Postoperative complications, neurologic outcomes, treatment delivery, and survival were treated as outcomes or downstream variables. The study was designed to evaluate associations and the construct validity of the surgical-intensity classification. It was not designed as a prediction model study and did not attempt to estimate a causal treatment effect. Because the study included the complete eligible cohort within a fixed study window, no a priori sample size calculation was performed. Survival follow-up continued until the administrative censor date of 6 July 2026. The study was approved by the Institutional Ethics Committee of Basaksehir Cam and Sakura City Hospital (approval no. 361; 16 July 2026). The requirement for informed consent was waived because only de-identified routinely collected data were analyzed. The manuscript was prepared in accordance with the STROBE and RECORD recommendations for observational studies using routinely collected health data [31,32].

### 2.2. Patient Selection and Index Treatment Episode

**Figure 1:**
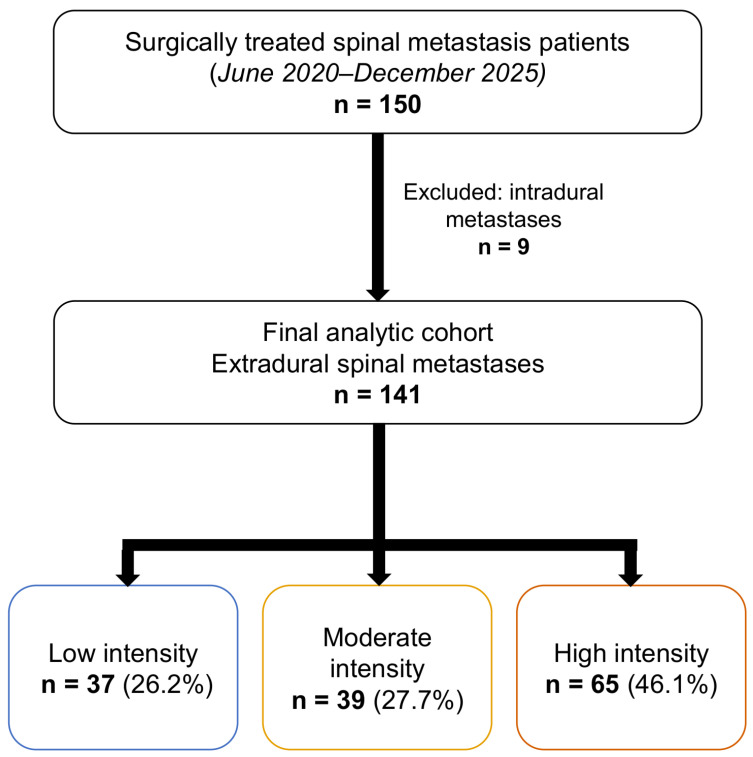
Cohort derivation. The source cohort comprised 150 patients with spinal metastases who were surgically treated. Nine patients with intradural metastases were excluded, leaving 141 patients with extradural spinal metastases. The final cohort was classified as low-, moderate-, or high-intensity surgery.

The source cohort comprised 150 adults who received operative treatment for spinal metastatic disease during the study period. Eligibility required an age of at least 18 years, an operation directed at metastatic involvement of the spine, and sufficient operative documentation to define the index treatment episode and assign surgical intensity. Nine patients with intradural metastases were excluded because intradural disease differs from extradural metastasis in operative anatomy, stabilization requirements, surgical goals, postoperative pathways, and prognosis. The final analytic cohort therefore included 141 patients with extradural spinal metastases (Figure 1). Patients with single-level or multilevel extradural disease, hematologic tumors involving the spine, or a primary tumor that was not yet known before surgery remained eligible when the other criteria were met.

Each patient contributed only the first eligible surgical treatment episode during the study window. The index episode was defined as the complete operative strategy selected for the presenting extradural spinal metastasis. When anterior and posterior procedures were planned as stages of the same treatment strategy, the stages were treated as one index episode; their operative components and total operative duration were combined for the surgical-intensity and SMII assessments. An unplanned reoperation for a complication, local recurrence, or later disease progression was not added to the index exposure and did not create a second study observation. Such procedures were recorded as postoperative outcomes. Patients with primary spinal tumors, nonmetastatic spinal disease, a missing index-operation date, or inadequate operative detail for intensity classification were not eligible. Stand-alone percutaneous vertebroplasty or kyphoplasty performed without decompression or tumor surgery was also excluded. No additional patients from the 150-patient source cohort were excluded beyond the nine intradural metastasis cases.

### 2.3. Clinical, Laboratory, Oncologic, and Survival Variables

Demographic, functional, oncologic, neurologic, imaging, operative, laboratory, and survival data were abstracted from the institutional record. Demographic variables were age, sex, and body mass index at the index admission. Clinical reserve was described using the American Society of Anesthesiologists class, Eastern Cooperative Oncology Group (ECOG) performance status, and metastatic spinal tumor frailty index. Oncologic variables included whether the primary tumor was known before surgery, the final primary tumor category, visceral metastasis, and the number of vertebral levels involved. Neurologic status was recorded from the last documented preoperative examination and included Frankel grade and ambulatory status. Mechanical instability and epidural compression were described with SINS and Bilsky grade, as detailed in Section 2.4. Operative variables included the components of the index treatment episode, index and total operative duration, transfusion, hospital length of stay, complications, and revision or reoperation. Preoperative laboratory values from the index surgical admission included hemoglobin, albumin, neutrophil-to-lymphocyte ratio, systemic immune-inflammation index, prognostic nutritional index, and the hemoglobin–albumin–lymphocyte–platelet score. Postoperative ambulatory status and Frankel change were assessed at postoperative days 10–14. Overall survival was measured from the date of the index operation to death. Patients who were alive at the administrative censor date were censored on that date.

Data were checked against the source record before analysis. No missing value was statistically imputed, and no patient was assigned a value solely from another correlated variable. Analyses used the available denominator for variables with incomplete documentation. Body mass index was available in 135 patients and visceral metastasis status in 137. SINS, Bilsky grades, SMII, the primary 90-day endpoint, vital status, and all covariates used in the primary Firth and Cox models were complete for the full cohort. Perioperative oncologic treatment variables were less consistently documented. They were therefore classified as documented, absent, or unknown and were reported descriptively using variable-specific denominators rather than imputed values.

### 2.4. Imaging Assessment

Two neurosurgeons experienced in spine oncology independently reviewed the preoperative imaging. They assessed the index level, thoracic location, number of involved segments, mechanical stability features, and epidural spinal cord compression while blinded to laboratory indices and survival outcomes. SINS was scored from the preoperative radiographic and clinical information and categorized as stable (0–6), potentially unstable (7–12), or unstable (13–18) [3,4]. Bilsky grade was determined from the axial T2-weighted MRI appearance at the level of greatest epidural compression. Grades 2 and 3 were grouped as high-grade epidural spinal cord compression [5]. Differences between the two reviewers were resolved by joint review and consensus. To assess reproducibility, 30 MRI examinations were selected for independent duplicate Bilsky grading. Exact agreement was observed in 27 of 30 examinations, and the weighted Cohen kappa was 0.734, indicating substantial agreement.

### 2.5. Tumor Biology Classification

Tumor biology was classified before outcome modeling using a prespecified, pragmatic Tomita-style growth framework [6]. The final primary tumor diagnosis was determined from the documented oncologic diagnosis and pathology record. A tumor that was not known before surgery was assigned to its final primary category when the postoperative diagnostic workup established the origin. The mapping covered all 141 patients and is shown in Supplementary Table S1. Breast, prostate, differentiated thyroid, multiple myeloma, and lymphoma tumors were placed in the slow-growth category. Renal cell, gynecologic, head and neck, and testicular or germ-cell tumors were placed in the moderate-growth category. Lung, colorectal, pancreatobiliary or hepatocellular, upper gastrointestinal, poorly differentiated neuroendocrine, melanoma, sarcoma, and anaplastic or mixed thyroid tumors were placed in the rapid-growth category. For the primary models, a binary aggressive solid/unknown variable grouped rapid-growth solid tumors and any unresolved unknown primary disease together. The reference category consisted of hematologic tumors and slow- or moderate-growth solid tumors. This binary grouping was chosen to preserve a clinically meaningful contrast while limiting the number of parameters in models with 29 deaths by 90 days. It was an analytic tumor biology variable rather than a new prognostic score and was not intended to replace tumor-specific oncologic assessment. Sensitivity analyses used the ordinal growth category, rapid versus non-rapid biology, and restriction to solid tumors to examine whether the findings depended on this binary definition.

### 2.6. Surgical-Intensity Classification

**Figure 2:**
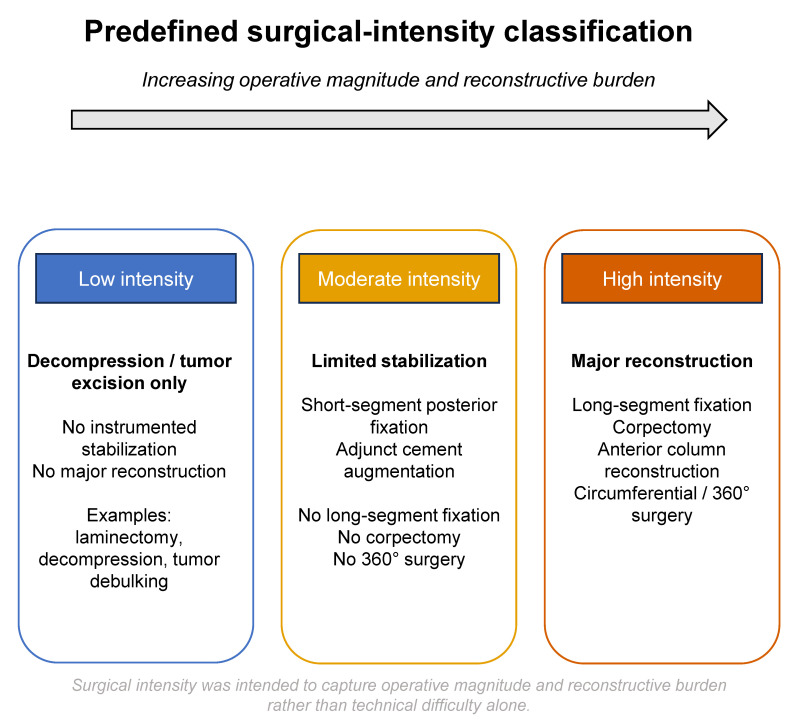
Predefined surgical-intensity classification. Low-intensity surgery comprised decompression or tumor excision without instrumented stabilization or major reconstruction. Moderate-intensity surgery comprised limited stabilization, short-segment posterior fixation, and/or adjunct cement augmentation without a high-intensity feature. High-intensity surgery comprised long-segment fixation, corpectomy, anterior column reconstruction, circumferential/360° surgery, or combinations of these features. Adjunct cement augmentation did not include stand-alone augmentation-only procedures.

The primary exposure was surgical intensity, classified a priori as low, moderate, or high from the operative strategy (Figure 2). Low-intensity surgery comprised decompression or tumor excision without instrumented stabilization or major reconstruction. Moderate-intensity surgery comprised decompression or tumor surgery with limited stabilization, short-segment posterior fixation, and/or adjunct cement augmentation, provided that no high-intensity feature was present. High-intensity surgery comprised long-segment fixation, corpectomy, anterior column reconstruction, circumferential/360° surgery, or combinations of these features. Cement augmentation denoted cement use as an adjunct to decompression or tumor surgery, including cement-augmented fixation or vertebroplasty/kyphoplasty performed additionally to decompression; stand-alone augmentation-only procedures were excluded. The classification was intended to capture overall operative magnitude and reconstructive burden rather than technical difficulty alone.

### 2.7. Spinal Metastasis Invasiveness Index

The original SMII was retrospectively calculated for every patient as a secondary comparator for convergent construct validity [23]. Component-level data included posterior and anterior decompression, total or partial corpectomy, pediculectomy, posterior instrumentation, anterior support, vertebroplasty, tumor vascularity, and preoperative embolization according to the published scoring system. For planned staged treatment episodes, components were summed across the stages belonging to that index strategy. Hypervascular status was assigned from the documented primary tumor type using the original SMII tumor-type definitions. No patient had documented preoperative embolization. SMII was treated as a continuous score, was calculated independently of the predefined intensity classification, and did not alter any intensity assignment. It was not entered simultaneously with surgical intensity in the survival models because both are overlapping procedure-derived measures.

### 2.8. Outcomes and Postoperative Survival Thresholds

The primary outcome was 90-day mortality. Secondary outcomes included 30-, 180-, and 365-day mortality; overall survival; postoperative ambulatory status; Frankel improvement or worsening; complications; revision/reoperation; operative duration; transfusion; and length of stay. Neurologic outcomes were evaluated at postoperative days 10–14.

Postoperative survival beyond 30, 90, 180, and 365 days was summarized as prespecified descriptive landmarks. These author-selected thresholds were not treated as validated outcome measures and were not interpreted as evidence of surgical appropriateness, quality-of-life benefit, or goal attainment. All patients were assessable at 30 and 90 days. At 180 and 365 days, patients were assessable if they died before the threshold or had undergone a follow-up beyond it; patients censored earlier were excluded from the threshold-specific denominator.

### 2.9. Perioperative Oncologic Treatment Context

Available radiotherapy and systemic treatment data were extracted as ancillary descriptive variables. Status was categorized as documented, absent, or unknown, and treatment timing as <30 days, 30–90 days, >90 days, or unknown when available. Postoperative radiotherapy and systemic therapy were not included as baseline adjustment covariates because they occur after surgical selection and are conditioned on postoperative survival, wound recovery, neurologic status, oncology access, and systemic disease trajectory. Treating them as baseline covariates could introduce immortal-time and treatment-selection bias.

### 2.10. Confounding Structure and Mediator Handling

Surgical intensity was not treated as a randomized or causal exposure. Mechanical instability, epidural compression, disease anatomy, tumor biology, expected survival, systemic reserve, neurologic status, patient goals, and surgeon judgment can influence the selected operation. ECOG status, tumor biology, albumin, visceral metastasis, frailty, and neurologic status may also influence survival. By contrast, operative duration, transfusion, length of stay, and complications occur after surgical selection and may mediate perioperative burden. These downstream variables were therefore used to evaluate convergent validity and perioperative consequences but were not entered as baseline confounders in the primary survival models.

### 2.11. Statistical Analysis

Continuous variables were summarized as medians with interquartile ranges and categorical variables as counts with percentages. Comparisons across intensity groups used Kruskal–Wallis tests for continuous variables and chi-square or Fisher exact tests for categorical variables, as appropriate. SMII distributions were compared with the Kruskal–Wallis test; the monotonic association between ordered intensity category and SMII was evaluated with Spearman rank correlation. Exploratory pairwise Wilcoxon comparisons used Holm adjustment.

Kaplan–Meier methods estimated overall survival by intensity group, and Cox proportional-hazards regression evaluated adjusted associations with overall survival. The proportional-hazards assumption was assessed using scaled Schoenfeld residuals [33]. Because 29 deaths occurred by 90 days, Firth penalized logistic regression was used for the primary binary endpoint [34,35]. The prespecified primary models included surgical-intensity category, ECOG ≥ 3, albumin, and aggressive solid/unknown tumor biology. Sensitivity analyses compared high versus low/moderate intensity, restricted the cohort to SINS ≥ 7, varied the tumor biology specification, and used overlap weighting for the binary intensity comparison [36]. The study was not designed or presented as prediction model development. Two-sided *p* < 0.05 was considered statistically significant; effect estimates and confidence intervals were emphasized, and absence of statistical significance was not interpreted as equivalence.

### 2.12. Use of Generative Artificial Intelligence

During manuscript preparation and revision, AI-assisted language-editing tools (ChatGPT, GPT-5.6; OpenAI, San Francisco, CA, USA) were used to improve clarity, organization, and readability. The authors independently verified all source data, analyses, references, interpretations, and conclusions and take full responsibility for the manuscript.

## 3. Results

### 3.1. Cohort and Baseline Characteristics

Of 150 surgically treated patients with spinal metastases, 9 with intradural metastases were excluded, leaving 141 patients with extradural disease. Surgical intensity was low in 37 patients (26.2%), moderate in 39 (27.7%), and high in 65 (46.1%). During follow-up, 97 patients died. Overall 30-day mortality was 5.0% (7/141), and 90-day mortality was 20.6% (29/141).

Baseline characteristics are summarized in Table 1. The most common primary tumors were lung carcinoma (*n* = 44), multiple myeloma (*n* = 21), breast carcinoma (*n* = 11), prostate carcinoma (*n* = 10), pancreatobiliary/hepatocellular carcinoma (*n* = 10), renal cell carcinoma (*n* = 9), lymphoma (*n* = 9), and colorectal carcinoma (*n* = 8). Tumor growth was classified as slow in 55 patients (39.0%), moderate in 16 (11.3%), and rapid in 70 (49.6%). ECOG ≥ 3 was present in 50 patients (35.5%), and visceral metastasis in 94 of 137 assessable patients (68.6%).

Age, BMI, ECOG ≥ 3, frailty, visceral metastasis, tumor-growth distribution, preoperative ambulatory status, Bilsky high-grade compression, albumin, NLR, SII, and HALP did not differ significantly across surgical-intensity groups. High-grade Bilsky compression was present in 28/37 (75.7%) low-intensity patients, 34/39 (87.2%) moderate-intensity patients, and 55/65 (84.6%) high-intensity patients (*p* = 0.366). SINS differed strongly. Median SINS was 8 [6–10], 9 [8–11], and 12 [10–14] in the low-, moderate-, and high-intensity groups, respectively (*p* < 0.001). Stable lesions accounted for 12/37 (32.4%), 3/39 (7.7%), and 1/65 (1.5%) of the three groups, whereas unstable lesions accounted for 4/37 (10.8%), 6/39 (15.4%), and 27/65 (41.5%), respectively (*p* < 0.001).

### 3.2. Surgical Intensity, SMII, and Perioperative Burden

**Figure 3:**
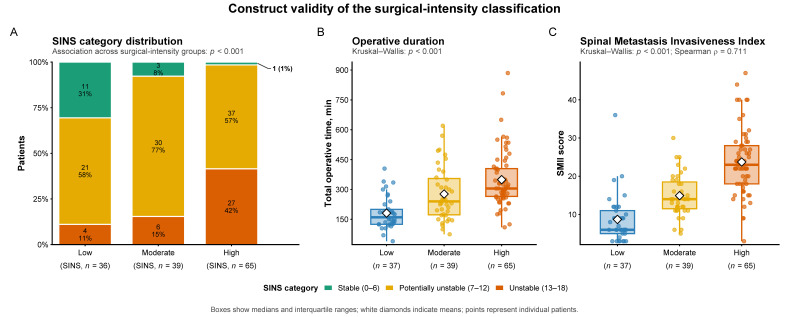
Construct validity of the surgical-intensity classification. (**A**) SINS category distribution across intensity groups. (**B**) Total operative duration. (**C**) SMII distribution. Boxes show medians and interquartile ranges, white diamonds indicate means, and points represent individual patients. SINS category differed across intensity groups (*p* < 0.001). Operative duration and SMII increased progressively (both *p* < 0.001), and ordered intensity category correlated strongly with SMII (Spearman ρ = 0.711, *p* < 0.001). SINS, Spinal Instability Neoplastic Score; SMII, Spinal Metastasis Invasiveness Index.

The operative features defining the three intensity categories are summarized in Table 2. High-intensity surgery was used in 27 of 37 unstable lesions (73.0%), whereas stable lesions were concentrated in the low-intensity group (Figure 3A). Cement augmentation was used only as an adjunct within surgically treated cases and occurred in seven moderate- and four high-intensity procedures.

SMII was calculable for all 141 patients and increased progressively across the predefined intensity groups. Median SMII was 6 [5–11] after low-intensity surgery, 14 [11.5–18.5] after moderate-intensity surgery, and 23 [18–28] after high-intensity surgery (Kruskal–Wallis H = 70.91, *p* < 0.001). Corresponding mean values were 8.70 ± 6.33, 14.92 ± 5.50, and 23.74 ± 8.53. Ordered intensity category showed a strong positive correlation with SMII (Spearman ρ = 0.711, *p* < 0.001), supporting convergent construct validity while confirming that the tiered classification and component-based score were related but nonidentical measures (Figure 3C; Supplementary Table S2).

Other measures of perioperative burden showed the same gradient. Median total operative duration was 160 [125–200], 240 [170–360], and 305 [265–405] minutes across low-, moderate-, and high-intensity surgery groups (*p* < 0.001; Figure 3B). Transfusion was required in 37.8%, 51.3%, and 69.2% of patients (*p* = 0.007), and median length of stay was 3 [2–7], 4 [3–7], and 6 [4–8] days (*p* = 0.003), respectively. Overall complication and revision/reoperation rates did not differ significantly.

### 3.3. Postoperative Survival Thresholds and Neurologic Outcomes

**Figure 4:**
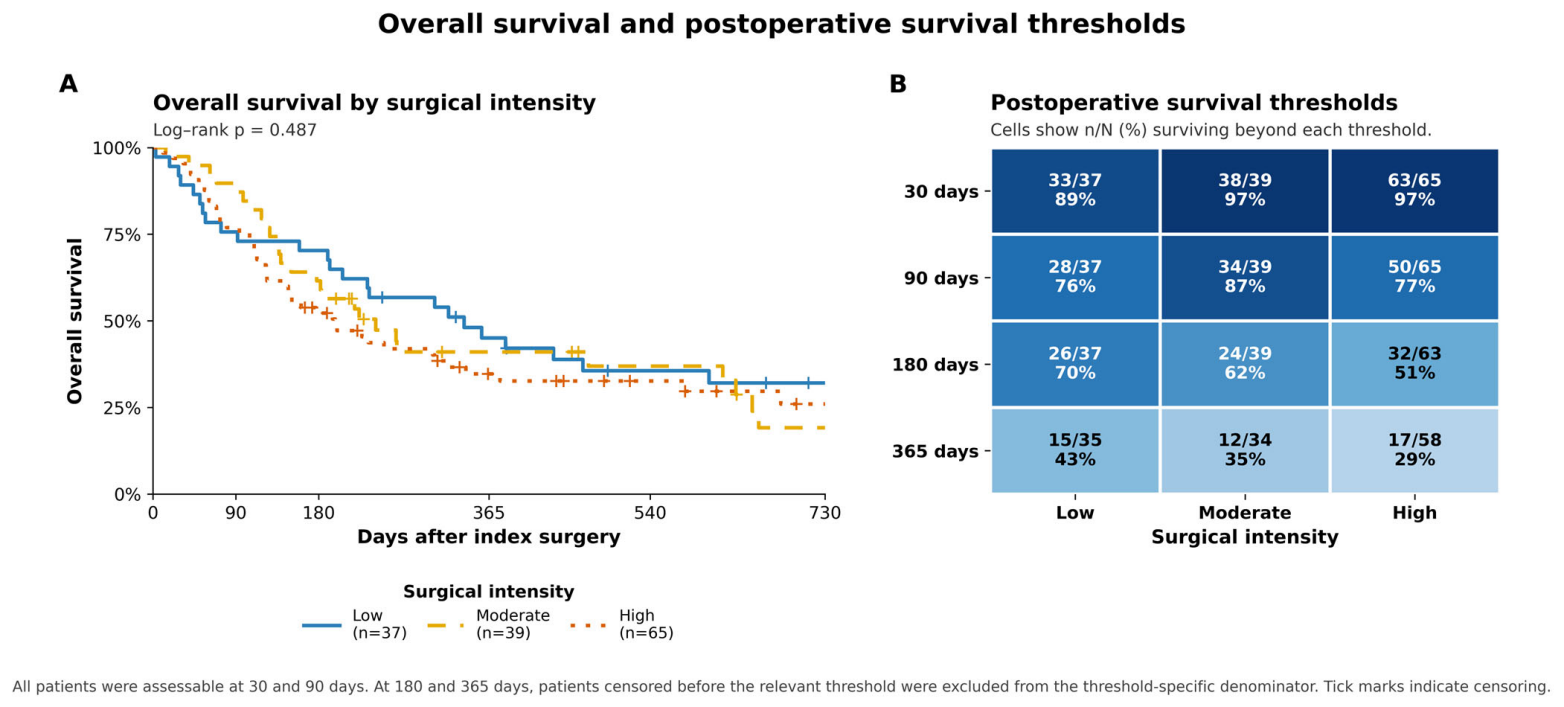
Overall survival and postoperative survival thresholds. (**A**) Kaplan–Meier overall survival curves by surgical-intensity group; tick marks indicate censoring. The curves did not differ significantly (log-rank *p* = 0.487). (**B**) Numbers and percentages of threshold-assessable patients surviving beyond 30, 90, 180, and 365 days. All patients were assessable at 30 and 90 days. At 180 and 365 days, patients censored before the relevant threshold were excluded from that threshold-specific denominator.

Survival outcomes are presented in Table 3 and Figure 4. Ninety-day mortality was 24.3% after low-intensity surgery, 12.8% after moderate-intensity surgery, and 23.1% after high-intensity surgery (*p* = 0.367). Thirty-, 180-, and 365-day mortality likewise did not differ significantly across groups.

Kaplan–Meier median survival was 338 days (95% CI 206-not estimable) after low-intensity surgery, 242 days (178–651) after moderate-intensity surgery, and 198 days (142–340) after high-intensity surgery. The curves did not differ significantly (log-rank *p* = 0.487). Among high-intensity patients, 63/65 (96.9%) survived beyond 30 days and 50/65 (76.9%) beyond 90 days. Among threshold-assessable high-intensity patients, 32/63 (50.8%) survived beyond 180 days and 17/58 (29.3%) beyond 365 days.

Postoperative ambulation at days 10–14 was similar across groups: 78.4% after low-intensity surgery, 76.9% after moderate-intensity surgery, and 78.5% after high-intensity surgery (*p* > 0.999). Frankel improvement occurred in 32.4%, 30.8%, and 20.0%, respectively (*p* = 0.293); worsening was uncommon (*p* = 0.353). Neurologic comparisons were exploratory because operations were selected for mechanical and reconstructive indications as well as neural compression.

### 3.4. Perioperative Oncologic Treatment Context

Available oncologic treatment data are summarized in Supplementary Table S3. Preoperative index-level radiotherapy was documented in 12 of 134 patients with known status (9.0%), any preoperative radiotherapy in 39/138 (28.3%), and preoperative systemic therapy in 47/137 (34.3%).

Postoperative radiotherapy status was known in 115 patients, of whom 64 (55.7%) received treatment. Postoperative systemic-therapy status was known in 115 patients, of whom 84 (73.0%) received treatment. Among 110 patients with both postoperative modalities documented, 91 (82.7%) received radiotherapy, systemic therapy, or both. These variables were retained as descriptive context and were not used to infer treatment continuity or causal mediation.

### 3.5. Primary and Sensitivity Models

**Figure 5:**
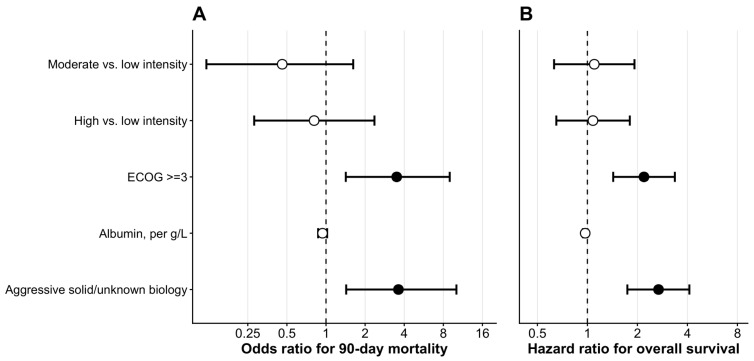
Adjusted early-mortality and overall survival models. (**A**) Primary Firth penalized logistic regression model for 90-day mortality. (**B**) Primary Cox proportional-hazards model for overall survival. Markers represent adjusted effect estimates and horizontal bars represent 95% confidence intervals; the dashed vertical line indicates the null value of 1. Open markers indicate estimates whose 95% confidence intervals include the null value, whereas filled black markers indicate statistically significant associations whose 95% confidence intervals exclude 1. Surgical intensity was not statistically associated with either outcome, whereas ECOG ≥ 3 and aggressive solid/unknown tumor biology were associated with worse outcomes. ECOG, Eastern Cooperative Oncology Group.

The primary adjusted models are shown in Table 4 and Figure 5. In the Firth model for 90-day mortality, moderate-intensity surgery had an adjusted OR of 0.46 (95% CI 0.12–1.62; *p* = 0.227) and high-intensity surgery had an OR of 0.81 (0.28–2.36; *p* = 0.694) relative to low intensity. ECOG ≥ 3 (OR 3.50, 1.42–8.96; *p* = 0.007) and aggressive solid/unknown biology (OR 3.61, 1.43–10.09; *p* = 0.006) were associated with higher 90-day mortality. Albumin showed a directionally protective but statistically nonsignificant association (OR 0.94 per g/L, 0.87–1.02; *p* = 0.126).

In the Cox model, moderate-intensity surgery had an adjusted HR of 1.10 (95% CI 0.63–1.92; *p* = 0.729) and high-intensity surgery had an HR of 1.08 (0.65–1.80; *p* = 0.759). ECOG ≥ 3 (HR 2.19, 1.43–3.36; *p* < 0.001) and aggressive solid/unknown biology (HR 2.68, 1.74–4.11; *p* < 0.001) were associated with worse overall survival. The proportional-hazards assumption was not violated (Supplementary Table S4).

Results remained concordant in the SINS ≥ 7 restriction: high intensity was not associated with 90-day mortality (OR 1.25, 0.50–3.18; *p* = 0.639) or overall survival (HR 0.98, 0.64–1.50; *p* = 0.937), whereas ECOG and tumor biology retained associations with outcome. In overlap-weighted analyses, high intensity was not statistically associated with 90-day mortality (OR 0.73, 0.32–1.64; *p* = 0.447) or overall survival (HR 0.95, 0.59–1.51; *p* = 0.821). Secondary threshold and tumor biology sensitivity analyses are provided in Supplementary Tables S5 and S6.

## 4. Discussion

This study evaluated a clinically defined three-tier classification of operative magnitude in 141 patients undergoing surgery for extradural spinal metastases. Three findings were central. First, the classification showed strong convergent validity: increasing intensity aligned with SINS-defined instability, SMII, operative duration, transfusion, and length of stay. Second, high-intensity surgery was not statistically associated with 90-day mortality or overall survival after adjustment, although the confidence intervals remained compatible with clinically meaningful benefit or harm and should not be interpreted as equivalence. Third, performance status and aggressive tumor biology showed more consistent associations with early mortality and overall survival than the selected operative tier.

The SMII analysis directly addresses whether the pragmatic intensity categories reflect an established measure of procedure-related burden. Median SMII increased from 6 to 14 to 23 across the low-, moderate-, and high-intensity groups, with a strong rank correlation. The measures are related but not interchangeable. SMII quantifies the weighted accumulation of specific operative components and vascular modifiers [23], whereas the three-tier classification communicates the overall reconstructive strategy. The observed within-group overlap is therefore expected, particularly for multilevel decompressions, planned staged procedures, and anatomically complex operations that may accumulate SMII points without meeting the predefined criteria for major reconstruction.

Modern spine oncology increasingly uses surgery as part of a hybrid treatment pathway rather than as an isolated definitive intervention. Separation surgery followed by stereotactic radiotherapy can provide durable local control while limiting the extent of tumor resection required around the spinal cord [26,27,28]. Recent matched comparative evidence supports this burden-reduction rationale. In patients with metastatic epidural spinal cord compression, separation surgery was associated with lower estimated blood loss and shorter operative duration than corpectomy with anterior reconstruction, without significant differences in hospitalization, complications, reoperations, or survival [37]. In this context, major surgery should be judged not only by immediate perioperative burden but also by whether it creates a survivable bridge to postoperative radiotherapy, systemic therapy, or both.

Mechanical instability was closely related to treatment magnitude. Nearly three quarters of unstable lesions underwent high-intensity surgery, while stable lesions were predominantly managed with lower-intensity strategies. This is clinically plausible: load-bearing failure, collapse, deformity, and extensive osseous destruction may require longer constructs or anterior-column reconstruction. At the same time, the potentially unstable SINS category included all three strategies, illustrating the real-world gray zone in which pain phenotype, collapse, alignment, epidural disease, tumor biology, expected survival, and surgeon judgment interact. The recent JASA prospective study similarly found that SINS informs but does not determine the need for instrumentation and that procedure choice must remain individualized within NOMS [30].

This selection structure makes crude comparisons of surgical magnitude vulnerable to confounding by indication. High-intensity surgery is generally chosen for more destructive mechanical disease; conversely, a limited operation may reflect either lesser instability or a deliberate effort to reduce burden in a patient with poor systemic reserve. Our SINS-restricted and overlap-weighted analyses were intended to probe this structure, but no retrospective analysis can fully reconstruct multidisciplinary judgment or unmeasured treatment goals. The neutral adjusted estimates should therefore be interpreted as an absence of statistical evidence for an independent association, not as proof that major reconstruction is uniformly safe or that intensity is irrelevant.

The numerically lower 1-year survival after high-intensity surgery is clinically important, but it should not be interpreted simplistically as a surgical harm signal. High-intensity surgery was used disproportionately for unstable SINS lesions and was accompanied by numerically higher rapid-growth and aggressive solid/unknown tumor biology, lower albumin, and lower HALP. Conversely, low-intensity surgery included more slow-growth tumors and slightly more hematologic malignancies. The apparent late survival disadvantage after high-intensity surgery likely reflects advanced mechanical disease occurring within an adverse systemic-oncologic context. This interpretation is supported by the neutral adjusted Cox, SINS-restricted, and overlap-weighted analyses.

The perioperative gradient was nevertheless clear. Operative duration, transfusion, length of stay, and SMII all increased with intensity. Large prospective registry data further demonstrate that postoperative morbidity is clinically consequential even when the selected operative strategy itself is not independently associated with survival. In the international MTRON cohort, postoperative adverse events were associated with longer hospitalization, poorer health-related quality of life, and reduced survival [38]. These findings support distinguishing the magnitude of the selected operation from the downstream adverse events through which perioperative burden may become clinically manifest. The findings also align with the purpose of SMII and with comparative studies in the literature showing that less invasive approaches can reduce blood loss and, in some settings, accelerate return to systemic therapy, although certainty is limited by heterogeneity and selection [23,25]. The clinical objective is not to minimize invasiveness indiscriminately. Rather, the least burdensome operation that reliably accomplishes decompression, stabilization, and the intended oncologic pathway should be selected.

Postoperative survival landmarks require careful interpretation. Most high-intensity patients survived beyond 30 days and approximately three quarters beyond 90 days, but fewer than one third of assessable patients survived beyond 1 year. These values provide concrete counseling information and describe the interval available for recovery and subsequent care. They do not establish that the operation was appropriate, that pain or quality of life improved, or that the patient achieved personal goals. Survival beyond a threshold is at most a necessary context for benefit, not a surrogate for benefit itself.

This distinction is particularly important because prospective spine-oncology cohorts have demonstrated durable improvements in condition-specific and generic patient-reported outcomes after selected surgery [29]. The JASA study likewise showed improvement across multiple patient-reported domains, while also demonstrating that SINS and instrumentation decisions are not reducible to a single threshold [30]. A further prospective multicenter study of 203 patients demonstrated postoperative improvement in neurologic function, performance status, pain, activities of daily living, and EQ-5D measures across spinal regions, although the magnitude and pattern of quality-of-life recovery varied by lesion level [39]. These findings further support prospective patient-centered assessment rather than treating postoperative survival landmarks as surrogates for surgical benefit. Our retrospective dataset did not contain validated pain, health-related quality-of-life, treatment-burden, or goal-attainment measures. Consequently, the present study cannot compare the patient-centered value of low-, moderate-, and high-intensity strategies. Future prospective work should combine operative invasiveness, pain and quality-of-life trajectories, functional recovery, wound outcomes, and time-dependent oncologic treatment delivery.

The associations of ECOG status and aggressive tumor biology with both 90-day mortality and overall survival are consistent with established prognostic literature [8,9,10,11,12,13,14,15,16,17,18,19,20,21]. Systemic reserve and disease trajectory remain central even when mechanical instability creates a compelling surgical indication. Albumin was directionally protective, and recent large-database work likewise identified nutritional and functional variables among determinants of short-term morbidity and mortality [22]. These findings support an integrated decision process: the mechanical problem defines what operation may be necessary, whereas tumor biology and systemic reserve influence whether the patient is likely to tolerate and live through the intended postoperative pathway.

The overall 90-day mortality of 20.6% and high 1-year mortality are consistent with contemporary metastatic spine surgery literature. Bhanot et al. reported 90-day and 1-year mortality rates of 29% and 59% in a population-based Ontario cohort [16]. Karhade et al. reported 90-day and 1-year mortality rates of 25.1% and 54.3% in the SORG machine-learning development cohort [11]. These benchmarks reinforce that short-horizon survival remains clinically relevant, even in the era of improved systemic therapies and stereotactic radiotherapy.

The study complements rather than replaces established prognostic systems. Tomita, Tokuhashi, NESMS, and SORG estimate survival or morbidity risk [6,7,8,9,10,11,12,13,14,15]. SMII characterizes component-level operative invasiveness [23]. The present tiered classification serves a narrower descriptive purpose: it organizes clinically recognizable reconstructive strategies and tests whether the selected magnitude independently tracks survival after accounting for major systemic and oncologic factors. It is not a prediction model and should not be used as a standalone treatment algorithm.

These findings support three practical principles. First, operative magnitude alone should not be used as an isolated reason to deny extensive stabilization or reconstruction when mechanical instability is compelling. Second, high-intensity surgery should not be interpreted as intrinsically safe; it produces greater operative time, transfusion need, and length of stay. Third, survival selection should remain anchored in tumor biology, performance status, and systemic reserve.

Strengths include a focused extradural cohort, complete ascertainment of the primary endpoint and primary-model variables, an a priori intensity schema, independent imaging review with Bilsky reliability assessment, explicit separation of baseline confounders from downstream mediators, Firth regression for sparse early mortality, SINS-restricted and overlap-weighted sensitivity analyses, formal proportional-hazards testing, and complete patient-level SMII calculation. The revised presentation also distinguishes operative features that define the tiers from independent measures used to evaluate convergent validity.

Several limitations should be emphasized. The retrospective single-center design introduces selection bias and limits generalizability. Operative strategy was chosen according to anatomy, mechanical instability, neurologic compression, systemic condition, expected survival, tumor biology, patient goals, and institutional practice; residual confounding is therefore unavoidable. SMII was reconstructed retrospectively, and component-level scoring of complex or staged operations may be vulnerable to documentation error despite source review. Molecular subtype, treatment line, disease-control state, radiation modality and dose, and time-dependent oncologic treatment decisions were incompletely captured. Validated patient-reported outcomes, pain relief, quality of life, and goal attainment were unavailable. The tiered classification has strong internal convergent validity but has not been externally validated. Finally, threshold-specific analyses at 180 and 365 days excluded patients censored before each landmark and were interpreted together with time-to-event analyses.

Clinically, operative magnitude should neither be ignored nor used in isolation. Major reconstruction imposes greater perioperative burden, but a mechanically inadequate limited procedure may fail to achieve the palliative objective. Conversely, anatomy alone should not drive extensive surgery when performance status, systemic reserve, and tumor biology indicate a very short survival horizon. Multidisciplinary decision-making should therefore ask two linked questions: what operation is required to solve the neurologic and mechanical problem, and does the patient’s systemic-oncologic profile support that operative burden?

## 5. Conclusions

In this cohort, the predefined three-tier surgical-intensity classification captured clinically meaningful differences in operative magnitude. Increasing intensity was strongly aligned with mechanical instability, SMII, operative duration, transfusion requirement, and length of hospitalization, supporting its use as a descriptive framework for communicating reconstructive and perioperative burden. After adjustment for performance status, albumin, and tumor biology, however, surgical-intensity category was not statistically associated with 90-day mortality or overall survival. These findings should not be interpreted as evidence of equivalence or as proof that extensive reconstruction is uniformly safe, because the confidence intervals remained compatible with clinically meaningful benefit or harm.

Poor performance status and aggressive solid/unknown tumor biology showed more consistent associations with survival than operative intensity. Clinical selection should therefore address two linked questions: What operation is required to achieve adequate decompression and mechanical stabilization? Does the patient’s systemic reserve, tumor biology, and anticipated postoperative oncologic pathway support that operative burden? The intensity classification should be regarded as a descriptive communication tool rather than a prognostic score or standalone treatment algorithm. Prospective multicenter validation incorporating pain relief, health-related quality of life, goal attainment, complications, functional recovery, and time to postoperative oncologic treatment is required to determine the patient-centered value of each intensity strategy.

## Figures and Tables

**Table 1:** Baseline clinical, oncologic, neurologic, imaging, and laboratory characteristics by surgical-intensity group.

Characteristic	Low Intensity (*n* = 37)	Moderate Intensity (*n* = 39)	High Intensity (*n* = 65)	*p* Value
**Demographic and systemic-reserve characteristics**				
Age, years	63 [55–69]	63 [54–70]	61 [49–69]	0.752
Male sex	27 (73%)	30 (77%)	41 (63%)	0.308
Body mass index, kg/m^2^	25.8 [23.1–28.7]	24.7 [23.2–27.7]	24.5 [22.5–28.4]	0.742
ASA class 3–4	22 (59%)	28 (72%)	43 (66%)	0.390
ECOG performance status ≥ 3	16 (43%)	13 (33%)	21 (32%)	0.529
MSTFI ≥ 3	15 (41%)	9 (23%)	21 (32%)	0.266
**Oncologic characteristics**				
Primary tumor known preoperatively	15 (41%)	20 (51%)	34 (52%)	0.483
Visceral metastasis	24/35 (69%)	26/38 (68%)	44/64 (69%)	>0.999
Tumor-growth category: slow	18 (49%)	13 (33%)	24 (37%)	0.561
Tumor-growth category: moderate	4 (11%)	6 (15%)	6 (9.2%)	
Tumor-growth category: rapid	15 (41%)	20 (51%)	35 (54%)	
Aggressive solid/unknown biology	15 (41%)	20 (51%)	35 (54%)	0.426
Hematologic tumor	9 (24%)	8 (21%)	13 (20%)	0.866
**Neurologic and imaging characteristics**				
Preoperative ambulatory status	23 (62%)	29 (74%)	46 (71%)	0.517
Vertebral levels involved: 1	8 (22%)	11 (28%)	16 (25%)	0.886
Vertebral levels involved: 2	8 (22%)	12 (31%)	16 (25%)	
Vertebral levels involved: 3	6 (16%)	3 (7.7%)	8 (12%)	
Vertebral levels involved: ≥4	15 (41%)	13 (33%)	25 (38%)	
SINS total	8 [6–10]	9 [8–11]	12 [10–14]	<0.001
SINS category: stable	12/37 (32%)	3/39 (7.7%)	1/65 (1.5%)	<0.001
SINS category: potentially unstable	21/37 (57%)	30/39 (77%)	37/65 (57%)	
SINS category: unstable	4/37 (11%)	6/39 (15%)	27/65 (42%)	
Bilsky grade 2–3	28/37 (76%)	34/39 (87%)	55/65 (85%)	0.366
**Laboratory reserve and inflammatory indices**				
Albumin, g/L	37 [31–42]	38 [33–41]	35 [32–40]	0.156
Albumin < 35 g/L	11 (30%)	12 (31%)	28 (43%)	0.313
Neutrophil-to-lymphocyte ratio	4.0 [2.0–6.4]	3.9 [2.5–5.4]	4.1 [2.7–7.3]	0.412
Systemic immune-inflammation index	987.8 [563.4–1319.2]	1035.5 [577.0–1657.7]	1181.4 [734.2–2659.7]	0.162
HALP score	2.7 [1.9–3.9]	2.7 [1.5–4.4]	2.2 [1.2–3.2]	0.082

Values are shown as *n*/*N* (%) or median [interquartile range]. Overall *p* values are shown on the first row of multilevel categorical variables. Continuous variables were compared with the Kruskal–Wallis test; categorical variables were compared with the chi-square or Fisher exact test, as appropriate. Available-case denominators were used for body mass index (*n* = 135) and visceral metastasis (*n* = 137). SINS, Bilsky grade, and all variables in the primary Firth and Cox models were complete for all 141 patients. ASA, American Society of Anesthesiologists; ECOG, Eastern Cooperative Oncology Group; HALP, hemoglobin–albumin–lymphocyte–platelet; MSTFI, metastatic spinal tumor frailty index; SINS, Spinal Instability Neoplastic Score.

**Table 2:** Operative features, convergent construct measures, and perioperative burden by surgical-intensity group.

Characteristic	Low Intensity (*n* = 37)	Moderate Intensity (*n* = 39)	High Intensity (*n* = 65)	*p* Value
**Operative features used to describe the surgical-intensity categories**				
Decompression component	37 (100%)	39 (100%)	58 (89%)	0.017
Any fixation	0 (0%)	36 (92%)	63 (97%)	<0.001
Short-segment fixation	0 (0%)	36 (92%)	2 (3.1%)	<0.001
Long-segment fixation	0 (0%)	0 (0%)	54 (83%)	<0.001
Corpectomy	0 (0%)	0 (0%)	19 (29%)	<0.001
Anterior reconstruction or approach	0 (0%)	0 (0%)	7 (11%)	0.017
Circumferential/360-degree surgery	0 (0%)	0 (0%)	4 (6.2%)	0.194
Adjunct cement augmentation	0 (0%)	7 (18%)	4 (6.2%)	0.009
**Convergent construct measures and perioperative burden**				
SMII score	6 [5–11]	14 [11.5–18.5]	23 [18–28]	<0.001
Index operative time, min	150 [125–185]	225 [170–300]	295 [235–355]	<0.001
Total operative time, min	160 [125–200]	240 [170–360]	305 [265–405]	<0.001
Any transfusion	14 (38%)	20 (51%)	45 (69%)	0.007
Length of stay, days	3 [2–7]	4 [3–7]	6 [4–8]	0.003
Any complication	14 (38%)	14 (36%)	25 (38%)	0.975
Any revision/reoperation	7 (19%)	13 (33%)	18 (28%)	0.372

Values are shown as *n* (%) or median [interquartile range]. Surgical-intensity categories were assigned from the operative strategy independently of the subsequently calculated SMII and were not revised on the basis of SMII values. For an index treatment episode comprising more than one operative stage, total operative time and SMII incorporated the recorded components across stages. The upper block lists operative descriptors used to define the categories; the lower block presents convergent measures of procedure-related burden. Adjunct cement augmentation denotes cement use with decompression/tumor surgery and excludes stand-alone vertebral augmentation-only procedures. SMII, Spinal Metastasis Invasiveness Index.

**Table 3:** Postoperative survival and neurologic outcomes by surgical-intensity group.

Outcome	Low Intensity (*n* = 37)	Moderate Intensity (*n* = 39)	High Intensity (*n* = 65)	*p* Value
**Survival outcomes**				
Deaths during follow-up	24/37 (64.9%)	28/39 (71.8%)	45/65 (69.2%)	-
Kaplan–Meier median survival, days (95% CI)	338 (206-NE)	242 (178–651)	198 (142–340)	0.487
30-day mortality	4/37 (10.8%)	1/39 (2.6%)	2/65 (3.1%)	0.227
90-day mortality	9/37 (24.3%)	5/39 (12.8%)	15/65 (23.1%)	0.367
180-day mortality	11/37 (29.7%)	15/39 (38.5%)	31/63 (49.2%)	0.150
365-day mortality	20/35 (57.1%)	22/34 (64.7%)	41/58 (70.7%)	0.411
**Neurologic outcomes at postoperative days 10–14**				
Postoperative ambulatory status	29/37 (78.4%)	30/39 (76.9%)	51/65 (78.5%)	>0.999
Frankel grade improvement	12/37 (32.4%)	12/39 (30.8%)	13/65 (20.0%)	0.293
Frankel grade worsening	1/37 (2.7%)	4/39 (10.3%)	2/65 (3.1%)	0.353

Values are shown as *n/N* (%) unless otherwise specified. The Kaplan–Meier *p* value is from the log-rank test. All patients were assessable for 30- and 90-day mortality. The 180-day outcome was assessable in 139 patients and the 365-day outcome in 127 patients; patients censored before a threshold were excluded from that threshold-specific denominator. Neurologic outcomes were evaluated at postoperative days 10–14. CI, confidence interval; NE, not estimable.

**Table 4:** Primary adjusted models for 90-day mortality and overall survival.

Variable	Adjusted Effect Estimate	95% CI	*p* Value
**Firth penalized logistic regression: 90-day mortality (** ***n*** ** = 141; 29 events)**			
Moderate vs. low intensity	Adjusted OR 0.46	0.12–1.62	0.227
High vs. low intensity	Adjusted OR 0.81	0.28–2.36	0.694
ECOG performance status ≥ 3	Adjusted OR 3.50	1.42–8.96	0.007
Albumin, per 1 g/L	Adjusted OR 0.94	0.87–1.02	0.126
Aggressive solid/unknown biology	Adjusted OR 3.61	1.43–10.09	0.006
**Cox proportional-hazards regression: overall survival (** ***n*** ** = 141; 97 deaths)**			
Moderate vs. low intensity	Adjusted HR 1.10	0.63–1.92	0.729
High vs. low intensity	Adjusted HR 1.08	0.65–1.80	0.759
ECOG performance status ≥ 3	Adjusted HR 2.19	1.43–3.36	<0.001
Albumin, per 1 g/L	Adjusted HR 0.97	0.94–1.00	0.074
Aggressive solid/unknown biology	Adjusted HR 2.68	1.74–4.11	<0.001

Low-intensity surgery was the reference category. The prespecified primary models included surgical-intensity category, ECOG performance status ≥ 3, albumin, and aggressive solid/unknown tumor biology. SMII was not entered with surgical intensity because both are procedure-derived, overlapping measures of operative magnitude. Operative time, transfusion, length of stay, and complications were not included as baseline confounders because they occur after surgical selection and may mediate perioperative burden. The proportional-hazards assumption was not violated (Supplementary Table S4). CI, confidence interval; ECOG, Eastern Cooperative Oncology Group; HR, hazard ratio; OR, odds ratio.

## Data Availability

The datasets generated and analyzed during the current study are not publicly available because of institutional and patient confidentiality restrictions but may be available from the corresponding author upon reasonable request and with appropriate institutional approval.

## Supplementary Materials

The following supporting information can be downloaded at https://www.mdpi.com/article/10.3390/cancers18152512/s1, Table S1, Primary tumor mapping for tumor-growth and aggressive-biology classifications; Table S2, SMII distribution and convergent construct-validity analyses; Table S3, Available perioperative oncologic treatment context; Table S4, Proportional-hazards assumption testing; Table S5, Secondary threshold Firth models; Table S6, Sensitivity analyses for 90-day mortality and overall survival. .
