# Supplementary material for: Surgical Intensity and Survival After Surgery for Extradural Spinal Metastases: Mechanical Instability, Tumor Biology, and Systemic Reserve
Source: Cancers (Basel). 2026 Aug 5;18(15):2512. doi: 10.3390/cancers18152512 (PMC13464836; doi:10.3390/cancers18152512)
Supplement: Supplementary file 1 [file cancers-18-02512-s001.zip › cancers-4474605-supplementary.pdf]

Supplementary Table S1. Primary tumor mapping for tumor-growth and aggressive-biology classifications.

Primary tumor category	n	Tomita-style growth category	Aggressive solid/unknown biology
Breast carcinoma	11	Slow	No
Prostate carcinoma	10	Slow	No
Thyroid carcinoma	4	Slow	No
Multiple myeloma	21	Slow	No
Lymphoma	9	Slow	No
Renal cell carcinoma	9	Moderate	No
Gynecologic carcinoma	2	Moderate	No
Head and neck carcinoma	2	Moderate	No
Testicular/germ cell tumor	3	Moderate	No
Lung carcinoma	44	Rapid	Yes
Colorectal carcinoma	8	Rapid	Yes
Pancreatobiliary/hepatocellular carcinoma	10	Rapid	Yes
Upper gastrointestinal carcinoma	3	Rapid	Yes
Poorly differentiated neuroendocrine carcinoma	1	Rapid	Yes
Melanoma	1	Rapid	Yes
Sarcoma	2	Rapid	Yes
Anaplastic/mixed thyroid carcinoma	1	Rapid	Yes

Tumor growth was grouped using a prespecified pragmatic Tomita-style survival-biology framework. The aggressive solid/unknown variable separated rapidly progressive solid or unknown-primary tumors from hematologic and more favorable solid-tumor categories while preserving model parsimony.

Supplementary Table S2. Available perioperative oncologic-treatment context.

Variable	Documented treatment	Unknown status	Timing or treatment composition
Preoperative index-level radiotherapy	12/134 (9.0%)	7	Not applicable
Any preoperative radiotherapy	39/138 (28.3%)	3	<30 d: 7; 30-90 d: 6; >90 d: 23; unknown timing: 3
Preoperative systemic therapy	47/137 (34.3%)	4	<30 d: 13; 30-90 d: 7; >90 d: 24; unknown timing: 3
Any preoperative radiotherapy or systemic therapy	53/137 (38.7%)	4 incomplete/unknown	Both: 32; RT only: 6; systemic only: 15
Postoperative index-level radiotherapy before POD 10-14	1/140 (0.7%)	1	Not applicable
Any postoperative radiotherapy	64/115 (55.7%)	26	<30 d: 17; 30-90 d: 29; >90 d: 11; unknown timing: 7
Postoperative systemic therapy	84/115 (73.0%)	26	<30 d: 37; 30-90 d: 37; >90 d: 4; unknown timing: 6
Any postoperative radiotherapy or systemic therapy	91/110 (82.7%)	31 incomplete/unknown	Both: 48; RT only: 11; systemic only: 32; neither documented: 19

Radiotherapy and systemic-therapy variables were summarized descriptively. Postoperative treatment variables were not included as baseline adjustment covariates because they occur after surgical selection and are conditioned on postoperative survival, wound recovery, neurologic status, oncology access, and systemic disease trajectory. POD, postoperative day; RT, radiotherapy.

Supplementary Table S3. Proportional-hazards assumption testing for Cox regression models.

Variable	Chi-square	df	p value
**Primary three-tier Cox model**			
Surgical intensity	3.070	2	0.215
ECOG performance status >=3	3.023	1	0.082
Albumin	2.831	1	0.092
Aggressive solid/unknown biology	0.333	1	0.564
Global	7.953	5	0.159
**Binary high-intensity Cox model**			
High-intensity surgery	0.744	1	0.388
ECOG performance status >=3	2.913	1	0.088
Albumin	2.846	1	0.092
Aggressive solid/unknown biology	0.336	1	0.562
Global	5.868	4	0.209

The proportional-hazards assumption was assessed using scaled Schoenfeld residuals. No variable-specific or global test indicated a statistically significant violation. ECOG, Eastern Cooperative Oncology Group.

Supplementary Table S4. Secondary threshold Firth models.

**Threshold outcome**	**High-intensity effect**	**ECOG >=3 effect**	**Aggressive solid/unknown effect**	**Assessable n/events**
30-day mortality	OR 0.48 (0.08-2.33); p=0.368	OR 18.45 (1.98-2453.15); p=0.007	OR 1.92 (0.39-12.27); p=0.429	141/7
180-day mortality	OR 1.83 (0.87-3.88); p=0.110	OR 2.80 (1.29-6.21); p=0.009	OR 2.52 (1.22-5.28); p=0.013	139/57
365-day mortality	OR 1.41 (0.61-3.27); p=0.421	OR 2.54 (1.06-6.42); p=0.036	OR 4.12 (1.85-9.67); p<0.001	127/83

Each secondary Firth model included binary high-intensity surgery, ECOG performance status >=3, albumin, and aggressive solid/unknown biology. Albumin estimates are omitted from this abbreviated table because the purpose was to summarize the prespecified exposure and principal clinical covariates. Threshold denominators include patients who died before the threshold or had follow-up beyond it. ECOG, Eastern Cooperative Oncology Group; OR, odds ratio.

Supplementary Table S5. Sensitivity analyses for 90-day mortality and overall survival.

Outcome/model	Variable	Adjusted effect (95% CI)	p value
**Restriction to patients with SINS >=7**			
90-day mortality (Firth)	High vs. low/moderate intensity	OR 1.25 (0.50-3.18)	0.639
90-day mortality (Firth)	ECOG performance status >=3	OR 3.18 (1.24-8.40)	0.016
90-day mortality (Firth)	Aggressive solid/unknown biology	OR 3.61 (1.38-10.53)	0.008
Overall survival (Cox)	High vs. low/moderate intensity	HR 0.98 (0.64-1.50)	0.937
Overall survival (Cox)	ECOG performance status >=3	HR 2.11 (1.34-3.33)	0.001
Overall survival (Cox)	Aggressive solid/unknown biology	HR 2.67 (1.71-4.17)	<0.001
**Overlap-weighted comparison of high vs. low/moderate intensity**			
90-day mortality	High vs. low/moderate intensity	OR 0.73 (0.32-1.64)	0.447
Overall survival	High vs. low/moderate intensity	HR 0.95 (0.59-1.51)	0.821

SINS-restricted models evaluated patients with potentially unstable or unstable lesions. Overlap weighting was used as a sensitivity analysis for the binary high-intensity comparison. CI, confidence interval; ECOG, Eastern Cooperative Oncology Group; HR, hazard ratio; OR, odds ratio; SINS, Spinal Instability Neoplastic Score.

Supplementary Table S6. SMII distribution and convergent construct-validity analyses.

Analysis/group	n	Mean +/- SD or statistic	Median [IQR]	Range	p value
**SMII distribution by surgical-intensity group**					
Low intensity	37	8.70 +/- 6.33	6 [5-11]	3-36	
Moderate intensity	39	14.92 +/- 5.50	14 [11.5-18.5]	5-30	
High intensity	65	23.74 +/- 8.53	23 [18-28]	3-47	
**Overall and pairwise analyses**					
Kruskal-Wallis test	141	H=70.905	-	-	<0.001
Ordered intensity vs. SMII	141	Spearman rho=0.711	-	-	<0.001
Low vs. moderate	-	Holm-adjusted pairwise Wilcoxon	-	-	1.0 x 10^-6
Low vs. high	-	Holm-adjusted pairwise Wilcoxon	-	-	1.1 x 10^-12
Moderate vs. high	-	Holm-adjusted pairwise Wilcoxon	-	-	8.5 x 10^-8

SMII was calculable for all 141 patients and was treated as a continuous comparator for convergent construct validation. Fourteen patients (9.9%) had a hypervascular primary under the applied SMII tumor-type rule, and no preoperative embolization was documented. Pairwise comparisons were exploratory and adjusted using the Holm method. IQR, interquartile range; SD, standard deviation; SMII, Spinal Metastasis Invasiveness Index.
